# Supplementary material for: Intraoperative Hypertension Is Associated with Postoperative Acute Kidney Injury after Laparoscopic Surgery
Source: J Pers Med. 2023 Mar 17;13(3):541. doi: 10.3390/jpm13030541 (PMC10058414; doi:10.3390/jpm13030541)
Supplement: Supplementary file 1 [file jpm-13-00541-s001.zip › Supplementary Figures.zip!/Supplementary Figures/FigureS1_Approval for Scientific Research Project.pdf]

中南大学湘雅三医院伦理委员会

快速审查审批件

伦理批件号：快Ⅰ 22233

项目名称	择期腹部手术全身麻醉患者术后急性肾损伤的相关因素研究		
经费来源	国际合作课题□　国家级科研课题☑　院企合作课题□<br>医院科研课题□　研究者自发课题□　其他__________		
申办者	中南大学湘雅三医院	研究机构	中南大学湘雅三医院
研究科室	麻醉科	主要研究者	刘星
审查类别	初始审查☑　跟踪审查□　重审□		
审查方式	快速审查☑　会议审查□		
伦理委员会意见	经审核，同意按备案文件进行研究。<br><br>中南大学湘雅三医院伦理委员会（盖章）<br>2022年10月28日		
注意事项	1. 声明：本伦理委员会的职责、人员构成、运行和记录遵循国际ICH-GCP，《涉及人的生物医学研究伦理审查办法》、《医疗器械临床试验质量管理规范》以及其他中国相关法规。<br>2. 遵循原则：请在临床试验过程中严格遵循医学伦理原则，切实保障受试者的权益，研究过程中如有对临床研究方案、知情同意书、招募材料、给受试者的其他材料等的任何修改及变更，请及时报告伦理委员会，获得本伦理委员会的书面批准后方可实施。<br>3. 涉及人类遗传资源采集、收集、买卖、出口、出境的研究项目，须在获得中国人类遗传资源管理办公室的批件并在伦理委员会备案后开展研究。<br>4. 跟踪审查：本中心发生的严重不良事件及与受试者安全相关的事件，需及时报告伦理委员会，伦理委员会有权根据对事件的评估结果做出新的决定。申请人暂停、提前终止、完成临床研究时，请及时向伦理委员会提交相关报告及必要附件。<br>5. 联系方式：中南大学湘雅三医院伦理委员会<br>联系电话：0731-88618938<br>邮箱：xy3irb@163.com，xy3irbreview@163.com		

IRB of The Third Xiangya Hospital of Central South University
expedited approvals

No：快 I 22233

Project Title	The study on related factors of acute kidney injury in patients underwent selected abdominal surgery		
Funding Resource	International cooperation projects☐ National research projects☑<br>Academic-enterprise cooperation project☐ Hospital research projects☐<br>Researcher-initiated project☐ other:__________		
Sponsor	the Third Xiangya Hospital, Central South University	Research Institution	the Third Xiangya Hospital, Central South University
Research Department	Anesthesiology Department	Principal Investigator	Xing Liu
Review category	Initial review☑ Follow-up review☐ Retrial☐		
Review method	Expedited review☑ Convened review☐		
IRB opinion	After review, it was approved to conduct research.<br><br>IRB of The Third Xiangya Hospital of Central South University<br>Date:Oct. 28,2022		
points for attention	1. Statement: the responsibilities, personnel composition, operation, and records of this IRB shall comply with international ICH-GCP, 《ethical review measures for biomedical research involving human beings》 《quality management specification for clinical trials of medical devices》 and other relevant Chinese laws and regulations.<br>2. Follow the principle: Please strictly follow the medical ethics principle in the clinical trial process, and effectively protect the rights and interests of the subjects. In case of any modification and change to the clinical research scheme, informed consent, recruitment materials, other materials for the subjects, please report to the IRB in time and obtain the written approval of the IRB before implementation.<br>3. Research projects involving the gathering, collection, trading, exportation and exit of human genetic resources shall be carried out after obtaining the approval of China human genetic resources management office and filing with the IRB.<br>4. Follow up review: serious adverse events and events related to the safety of subjects in the center shall be reported to the IRB in a timely manner. The IRB has the right to make new decisions based on the evaluation results of the event. When the applicant suspends, terminates or completes the clinical study in advance, please submit the relevant report and necessary attachments to the IRB in time.<br>5.Contact information: IRB of Xiangya Third Hospital of Central South University<br>Tel: 0731-88618938<br>Email: xy3irb@163.com, xy3irbreview@163.com		
