# Supplementary material for: Intraoperative Hypertension Is Associated with Postoperative Acute Kidney Injury after Laparoscopic Surgery
Source: J Pers Med. 2023 Mar 17;13(3):541. doi: 10.3390/jpm13030541 (PMC10058414; doi:10.3390/jpm13030541)
Supplement: Supplementary file 1 [file jpm-13-00541-s001.zip › Supplementary Figures.zip!/Supplementary Figures/FigureS2.pdf]

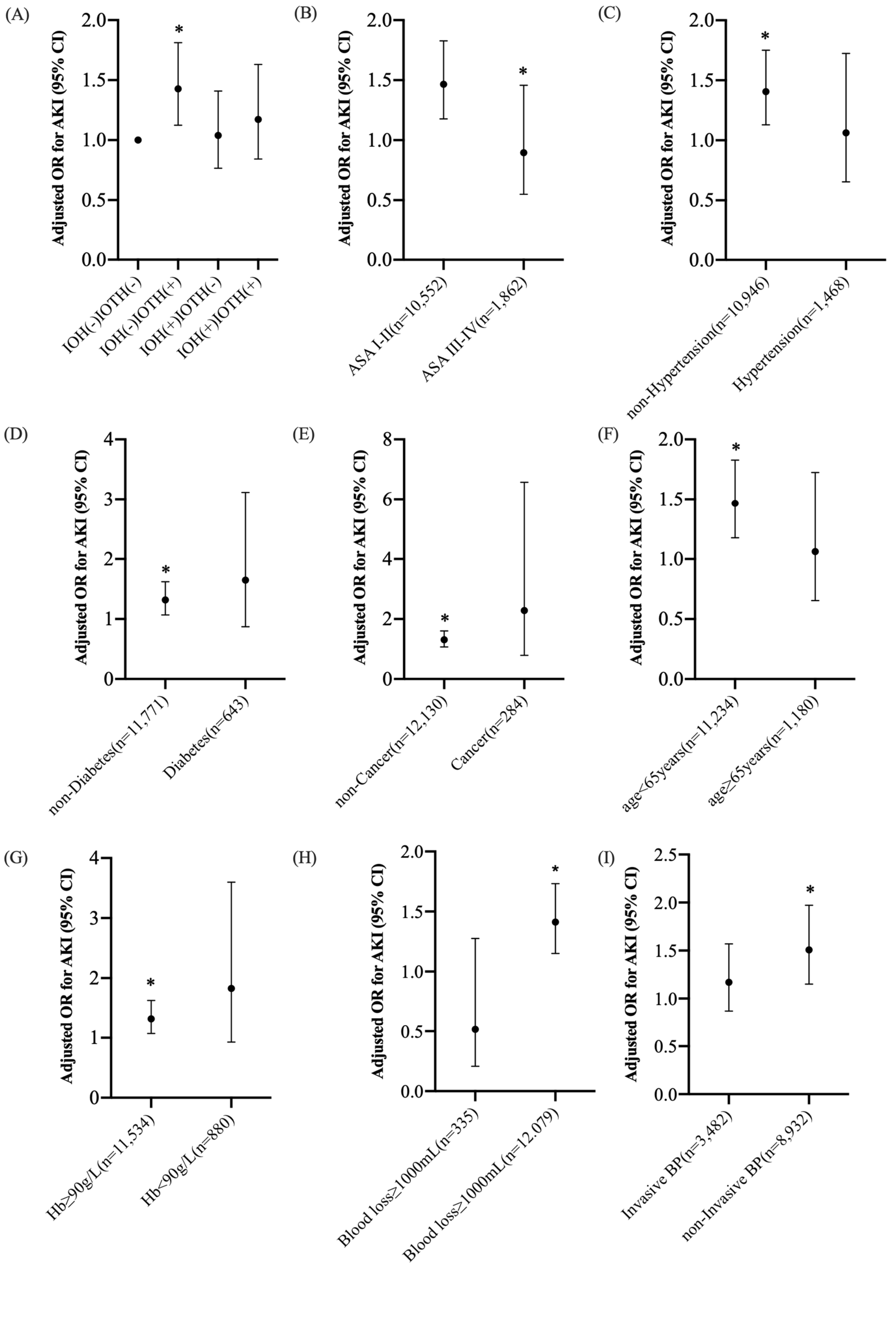
(A)
Adjusted OR for AKI (95% CI)
IOH(-)IOTH(-)
IOH(-)IOTH(+)
IOH(+)IOTH(-)
IOH(+)IOTH(+)
(B)
Adjusted OR for AKI (95% CI)
ASA I-II(n=10,552)
ASA III-IV(n=1,862)
(C)
Adjusted OR for AKI (95% CI)
non-Hypertension(n=10,946)
Hypertension(n=1,468)
(D)
Adjusted OR for AKI (95% CI)
non-Diabetes(n=11,771)
Diabetes(n=643)
(E)
Adjusted OR for AKI (95% CI)
non-Cancer(n=12,130)
Cancer(n=284)
(F)
Adjusted OR for AKI (95% CI)
age<65years(n=11,234)
age≥65years(n=1,180)
(G)
Adjusted OR for AKI (95% CI)
Hb≥90g/L(n=11,534)
Hb<90g/L(n=880)
(H)
Adjusted OR for AKI (95% CI)
Blood loss≥1000mL(n=335)
Blood loss≥1000mL(n=12.079)
(I)
Adjusted OR for AKI (95% CI)
Invasive BP(n=3,482)
non-Invasive BP(n=8,932)
